# Editorial: Advances in glycopeptide hydrogel for tissue engineering

**DOI:** 10.3389/fbioe.2026.1843053

**Published:** 2026-07-09

**Authors:** Jian Wang, Mengqin Zhou, Jinjian Huang, Yuhao Qiang, Zongan Li

**Affiliations:** 1 Research Institute of General Surgery, Jinling Hospital, School of Medicine, Nanjing University, Nanjing, China; 2 Department of Engineering, University of Maryland Eastern Shore, Princess Anne, MD, United States; 3 Jiangsu Key Laboratory of 3D Printing Equipment and Manufacturing, NARI School of Electrical and Automation Engineering, Nanjing Normal University, Nanjing, China

**Keywords:** biomaterials, dynamic hydrogel, extracellular matrix (ECM), glycopeptide hydrogel, tissue engineering

**FIGURE 1:**
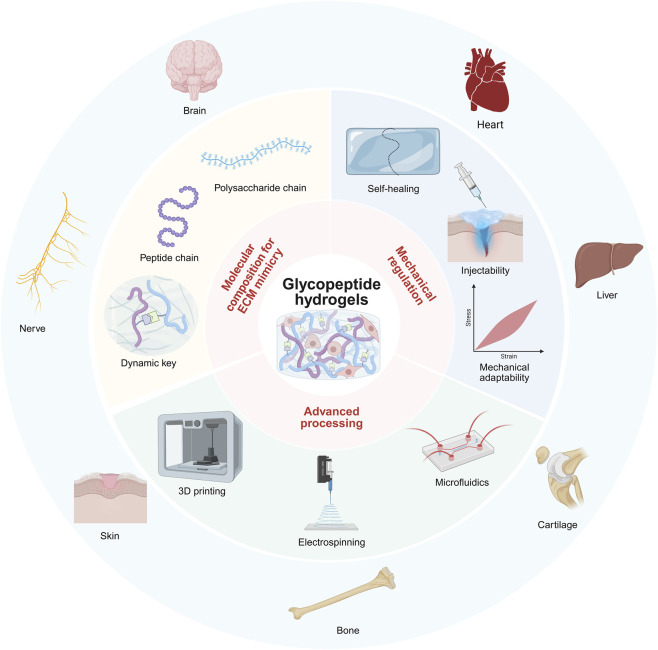
Overview of ECM-mimic molecular composition, mechanical characterization, manufacturing methods, and applications of glycopeptide hydrogels. Created with Biorender.com.

There exists a bidirectional interaction between cells and the surrounding extracellular matrix (ECM), driving increasingly stringent requirements for biomimetic matrices in the field of tissue engineering (Liu et al., 2026). However, conventional static cross-linked hydrogels face significant application bottlenecks due to their insufficient mechanical properties, lack of self-healing capability, and poor adaptability to dynamic biological environments. These materials struggle to precisely regulate degradation rates and exhibit low loading efficiency for bioactive molecules, which limits their broader application in precision medicine. To overcome these limitations, researchers have turned their attention to glycopeptide hydrogels—a novel class of multifunctional biomaterials constructed from polysaccharides and peptide chains via dynamic covalent bonds (e.g., Schiff base bonds, and boronate ester bonds) and supramolecular interactions (e.g., hydrogen bonding, π-π stacking, electrostatic interactions). Compared to traditional hydrogels, glycopeptide hydrogels demonstrate superior dynamic responsiveness, enhanced bioactivity, and excellent mechanical properties. This Research Topic focuses on three key dimensions of glycopeptide hydrogels—molecular design, mechanical regulation, and advanced processing—summarizing representative research progress and discussing their potential role and value in tissue engineering (Figure 1).

**FIGURE 2:**
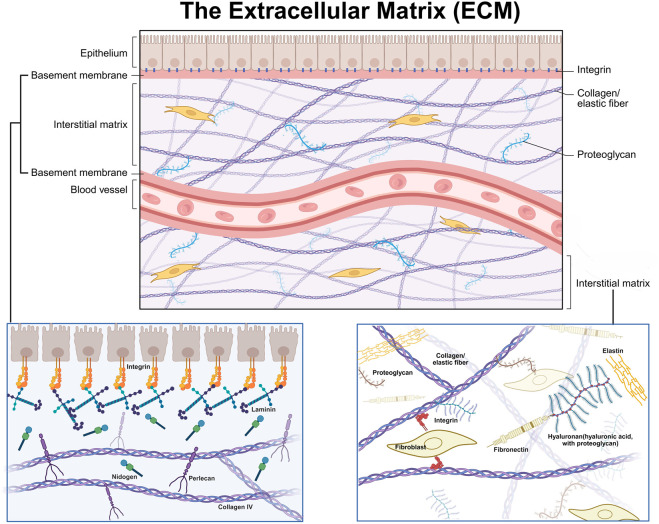
Schematic diagram of the hierarchical structure of glycopeptide components in the ECM. Created with Biorender.com.

The core of glycopeptide hydrogel molecular composition design lies in mimicking the chemical constituents and biological functions of the natural ECM (Figure 2), achieving structural biomimicry or metabolic microenvironment regulation through precise assembly of polysaccharides and peptides to promote tissue regeneration. As exemplified by the design strategy of constructing dynamic crosslinked networks through the introduction of reversible dynamic covalent bonds, glycopeptide hydrogels are endowed with precise control over their degradation behavior. Furthermore, such biomimetic mechanisms endow glycopeptide hydrogels with cell signaling characteristics. On one hand, peptide sequences (such as RGD) bind to integrins on the cell surface, activating mechanotransduction pathways such as focal adhesion kinase, thus providing a favorable environment for cell adhesion, proliferation, and signal transduction. On the other hand, sugar moieties (such as mannose) specifically bind to receptors on immune cell surfaces, triggering specific immune modulation. This material can also possess dynamic responsiveness, achieving matrix remodeling on demand through enzymatic degradation sites or utilizing dynamic bonds for the time-controlled release of growth factors. Consequently, this integrated design coordinates structural, mechanical, and biochemical signals to synergistically regulate cell.

Traditional single-component hydrogels struggle to fully provide the multidimensional biological signals required by the ECM. In contrast, glycopeptide hydrogels closely mimic the molecular composition of the native ECM, which forms the basis for their superior biological functionality. It has been elucidated that integrating fungal extracts with natural anti-inflammatory activity (primarily composed of polysaccharides) into a biomimetic hydrogel system can synergistically leverage the material’s physical barrier function and the pharmacological effects of the active components, enabling precise regulation of the inflammatory microenvironment in wounds (Arbab et al.). Guo et al. developed an injectable, self-healing glycopeptide hydrogels (OGA-CMCS) formed through a dynamic Schiff base reaction between oxidized glycyrrhizic acid (OGA) and carboxymethyl chitosan (CMCS). This hydrogel mimics the structure and dynamic microenvironment of native ECM glycoproteins, achieving anti-inflammatory and antibacterial effects in acute wounds, as well as inducing macrophage M2 polarization to promote tissue regeneration. This design strategy for glycopeptide hydrogels based on natural bioactive molecules achieves precise biomimicry of ECM structure and function at the molecular composition level, offering an ideal cellular support platform for tissue regeneration. Furthermore, the network structure of this hydrogel is precisely based on reversible Schiff base dynamic bonds, whose hydrolysis rate can be flexibly regulated through crosslinking density and the chemical microenvironment. It has been confirmed that by adjusting the degree of oxidation, a tunable degradation window ranging from several days to 2 weeks can be achieved, enabling the matching of degradation rate with the tissue regeneration process. This design strategy based on dynamic chemistry represents a key advantage of glycopeptide hydrogels over traditional statically crosslinked hydrogels, providing a molecular foundation for their programmed degradation and on-demand therapeutic application.

An ideal tissue engineering scaffold requires not only chemical biomimicry but also precise matching of the mechanical properties of native tissues to achieve physical regulation of cellular behavior. For instance, a study developed a hyaluronic acid-based double-network hydrogel that allows independent tuning of stiffness and viscoelasticity, thereby enabling investigation of how tissue mechanics and immune cells (macrophages) involved in the fibrotic process synergistically (Astrab et al., 2025; Zhou et al., 2026). Zhang et al. systematically elaborated on the design principles and functional optimization strategies for glycopeptide hydrogels, with particular emphasis on the pivotal role of dynamic crosslinking strategies in optimizing their mechanical performance. By modulating the dynamic covalent bonds and supramolecular interactions between sugars and peptides, the viscoelasticity, self-healing capability, and shear-thinning properties of the hydrogels were significantly enhanced. This dynamic mechanical behavior not only endows the hydrogels with excellent injectability and adaptability to irregular defects but also provides essential physical-mechanical cues for stem cell differentiation and tissue morphogenesis by mimicking the stress-relaxation behavior of the ECM. Current glycopeptide hydrogels still struggle to fully meet the complex mechanical demands of native soft tissues. Therefore, enhancing viscoelasticity represents a core challenge in achieving tissue biomimicry and dynamic adaptability.

In the construction of highly biomimetic and functionalized glycopeptide hydrogels, advanced processing technologies are key to achieving their precise structures and personalized applications. Han et al. and Kou et al. respectively demonstrated the enabling role of advanced processing technologies on the performance of glycopeptide hydrogels from different perspectives. The former focused on 3D printing technology, highlighting its capability to precisely fabricate glycopeptide hydrogels into 3D scaffolds with specific microporous structures. This enables personalized adaptation to spinal cord defects and guides the directional growth of nerve axons. The latter systematically introduced electrospinning technology, which processes glycopeptide materials into nanofiber membranes with high specific surface areas and porous structures. This mimics the fibrous topology of the native ECM, providing cells with physical and contact guidance while enabling the programmed release of various bioactive molecules. Furthermore, it can be integrated with technologies such as 3D printing and microfluidics to achieve more personalized and precise customization. Traditional preparation methods often struggle to simultaneously meet the requirements for fine control at the micro/macro scale and the demands of industrial-scale production. It is precisely the integration of advanced processing technologies that facilitates the construction of structurally and functionally integrated glycopeptide hydrogels, propelling them onto a practical pathway for tissue engineering applications.

In summary, glycopeptide hydrogels have emerged as a highly promising biomaterial platform in the field of tissue engineering due to their unique chemical, physical, and biological properties. The aforementioned research systematically demonstrates the significant potential of glycopeptide hydrogels in mimicking the ECM, regulating cell behavior, and promoting tissue regeneration, spanning from molecular design and mechanical modulation to advanced fabrication. These studies not only provide innovative solutions for various clinical challenges involving tissue regeneration but also deepen our understanding of biomimetic material design by elucidating the interaction mechanisms among materials, cells, and tissues. Unlike single-component hydrogels, the dynamic cross-linked network of glycopeptide systems allows for more convenient modulation of mechanical properties while integrating the immunomodulatory characteristics of polysaccharides with the targeting functions of peptides, thereby exhibiting a unique synergistic effect of dual “chemical-biological” activities. However, existing glycopeptide hydrogels still struggle to fully meet the complex mechanical demands of natural soft tissues, such as toughness, shear recovery, and stress relaxation. Further enhancement of viscoelastic properties is required to better achieve tissue biomimicry and dynamic adaptability. Additionally, decoding organ-specific matrix compositions and glycosylation patterns is necessary to guide the precise microenvironment-mimicking design of glycopeptide hydrogels. Nevertheless, it should not be overlooked that this multifunctional integration also entails greater design complexity, more intricate synthesis processes, and higher production costs. Striking a balance among these factors requires further exploration by researchers.

With their synergistic “glycan–peptide” molecular design, self-healing and responsive capabilities derived from reversible/dynamic networks, and the integration of structure and function achieved through mechanical tuning and advanced fabrication, glycopeptide hydrogels have demonstrated significant advantages and broad application potential in tissue engineering areas such as chronic wound healing, osteochondral repair, and neural regeneration (Figure 1). Specific contributions include: 1) at the molecular level, closer mimicry of the native ECM, providing sites for cell recognition and immune regulation; 2) at the material mechanical level, achieving mechanical matching with target tissues and regulating cell fate through various strategies; and 3) in fabrication processes, integration with advanced processing technologies such as electrospinning and 3D printing, enhancing clinical applicability and customization capabilities. Glycopeptide hydrogels are progressively evolving from an academic concept of biomimetic structures into a tunable, multifunctional, and scalable tissue engineering platform.

Despite the unique advantages of glycopeptide hydrogels in tissue engineering, their clinical translation still faces multiple challenges. Future research directions include a deep integration with 3D bioprinting technology to develop printable glycopeptide bioinks, enabling the precise construction of personalized biomimetic scaffolds. Overcoming clinical translation barriers involves achieving batch stability for large-scale production and long-term biosafety validation in large animals. Additionally, incorporating mechanistic studies on material–immune system interactions may shift the paradigm from passive mechanical matching to active immune regulation. Furthermore, to achieve spatiotemporal precise programming for drug release and material degradation, responsive design can incorporate multiple stimulus-responsive mechanisms (such as pH/ROS/enzymes). With the expansion of functional modules, precise matching of mechanical properties, and in-depth research into organ-specific microenvironments, glycopeptide hydrogels are expected to transition from structural biomimicry to functional regeneration, advancing personalized tissue repair and precision medicine to new heights.
